# Apical Hypertrophic Cardiomyopathy Prompting Aneurysm, Thrombus, and Cardiac Arrest in a 56-Year-Old Female

**DOI:** 10.7759/cureus.26067

**Published:** 2022-06-18

**Authors:** Patrick Biskupski, Julieta Osella, Aditya Bhaskaran, Andrii Maryniak, Mahmoud Khalil, Kenneth Ong

**Affiliations:** 1 Internal Medicine, Lincoln Medical Center, New York City, USA; 2 Internal Medicine, State University of New York Downstate Medical Center, Brooklyn, USA; 3 Cardiology, Lincoln Medical Center, New York City, USA

**Keywords:** thrombosis, aneurysm, cardiac arrest, ventricular tachycardia, hypertrophic cardiomyopathy

## Abstract

Hypertrophic cardiomyopathy (HCM) is the most prevalent genetic cardiac disease while apical hypertrophic cardiomyopathy (apHCM) is a rare subset of HCM. The significance of this case report is to present apHCM, its chronological course, and its association with left ventricular aneurysm, thrombosis, and cardiac arrest. We present the case of a 56-year-old female with a past medical history of apHCM who was admitted for substernal chest pain, developed a ventricular storm (VT), and subsequently suffered cardiac arrest; resuscitation of spontaneous circulation (ROSC) was eventually achieved after 10 minutes. It was initially thought that her arrhythmia and hemodynamic decompensation were purely secondary to cocaine use at a party six hours prior to her presentation. During hospitalization, cardiac magnetic resonance imaging demonstrated a severe apHCM apical aneurysm, thrombosis, and a re-entrant circuit as a likely cause of this patient’s decompensation and eventual cardiac arrest. After several days of hemodynamic stability and decreased dependence on intravenous antiarrhythmic medication infusions, she was extubated and transitioned to oral amiodarone and beta-blocker therapy with the implantation of a cardioverter-defibrillator (ICD). In this case, we analyze the continuum of apHCM, a rare subset of HCM once thought to be benign but with the emergence of complications, including aneurysm, thrombus formation, resistant ventricular tachycardia, and cardiac arrest. Recognition and management of apHCM with medical and/or surgical intervention are therefore critical to prevent the aforementioned sequela.

## Introduction

Hypertrophic cardiomyopathy (HCM) is the most prevalent genetic cardiac disease. It is diagnosed with cardiac imaging and can be divided into four subtypes: sigmoidal, reverse curve, neutral, and apical hypertrophic cardiomyopathy (apHCM) [1]. ApHCM or Yamaguchi syndrome, first described in 1976 by Hiroshi Yamaguchi [2], is a rare subset of HCM affecting the left ventricular apex, with no effect on the left ventricular outflow tract (LVOT). Historically acknowledged as a relatively benign structural heart disease, apHCM has resurfaced as a hallmark for arrhythmia, aneurysm, and thrombosis. It has an all-cause mortality rate greater than classic hypertrophic cardiomyopathy [3]. In this case report, we hope to increase awareness of this unfamiliar variant of hypertrophic cardiomyopathy and its clinical sequela.

The typical clinical features of apHCM include, palpable and audible fourth heart sound, indicating impaired LV relaxation, giant negative T waves on ECG, particularly in left precordial leads, associated apical wall motion abnormalities, such as hypokinesis, aneurysm formation, and spade-like configuration of LV in end-diastole on imaging. The diagnosis of apHCM requires imaging; options include echocardiography, left ventriculography, computed tomography, and, most accurately, cardiac magnetic resonance imaging (CMR) [3]. The differential diagnosis of apHCM comprises hypertrabeculation, non-compaction, mural thrombus, and/or endomyocardial fibrosis all of which can be distinguished by magnetic resonance imaging (MRI) using steady-state free precession (SSFP) imaging techniques and late gadolinium enhancement (LGE). Initial management is medical with first-line regimens including beta-blockers, improving midventricular obstruction and cavity obliteration (MVOCO), reducing symptomatic burden, and non-dihydropyridine calcium channels blockers as the second-line alternative. If medical management fails, surgical intervention is possible with septal or apical myomectomy [3]. This is the report of a 56-year-old woman with a left ventricular aneurysm, mural thrombus formation, and cardiac arrest associated with apical hypertrophic cardiomyopathy.

## Case presentation

**Figure 1:**
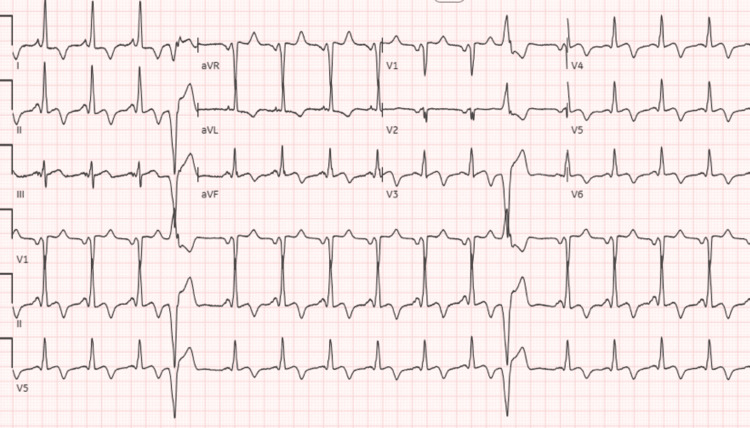
Initial EKG on presentation showing sinus rhythm with premature ventricular complexes and chronic T wave inversions

**Figure 2:**
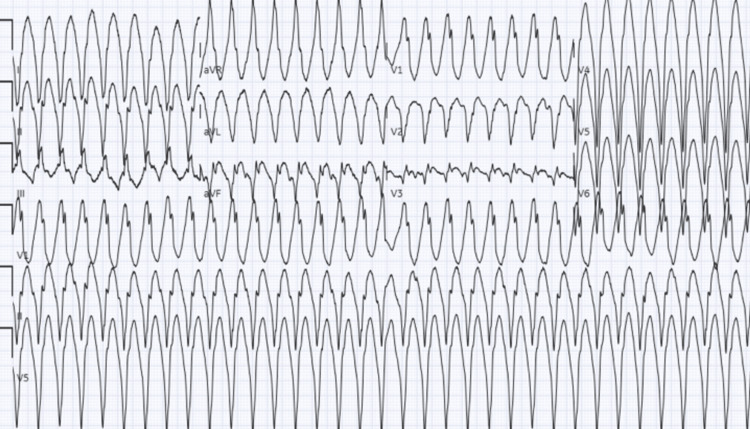
Repeat EKG while admitted showing monomorphic ventricular tachycardia

A 56-year-old female with a past medical history of apHCM, hypertension, hyperlipidemia, and prediabetes presented to the emergency department complaining of an intense substernal chest pain that radiated to the left upper extremity. She had associated dyspnea and experienced a syncopal episode lasting less than 30 seconds, reportedly having used cocaine at a party six hours prior to arrival. At the bedside, the patient was comfortable, in no acute distress, alert and oriented x 4, and following all commands. Cardiac auscultation yielded normal heart sounds, no murmurs, no rubs, no gallops, vital signs yielded a heart rate of approximately 80 beats per minute, and normotensive. No jugular venous distention or pedal edema was noted on further examination. Vesicular breath sounds were appreciated bilaterally throughout all lung fields and pulse oximetry provided a saturation of 98 percent on room air. Initial electrocardiography (EKG) showed a normal sinus rhythm (Figure 1). After arrival, her chest pain briefly subsided, returning with increasing severity and similar radiation to her left arm. Repeat EKG was performed and showed sustained monomorphic ventricular tachycardia (VT) (Figure 2). No evidence of the R on T phenomenon was identified on telemetry. Chemical cardioversion was initiated, and the patient received a 150 mg IV bolus of amiodarone twice and was subsequently started on a continuous infusion. VT persisted with resultant hemodynamic instability and thus electrical cardioversion was attempted. The patient was sedated and shocked with 100 joules, temporarily converting her rhythm to the normal sinus rhythm. VT returned despite two more shocks with increasing energy. In the setting of shock-resistant VT, the patient received a loading dose of lidocaine, was started on a continuous infusion, and metoprolol was added. She was intubated, mechanically ventilated, sedated with propofol, and transferred to an outside hospital with cardiac catheterization capabilities for a higher level of care. The patient consequently experienced a VT arrest in the tertiary care facility, requiring cardiopulmonary resuscitation (CPR), and return of spontaneous circulation (ROSC) was achieved after 10 minutes. After several days of hemodynamic stability, and decreased dependence on intravenous antiarrhythmic medication infusions, she was extubated, and transitioned to oral amiodarone and beta-blocker therapy with the implantation of an implantable cardioverter-defibrillator (ICD).

Diagnosis

**Figure 3:**
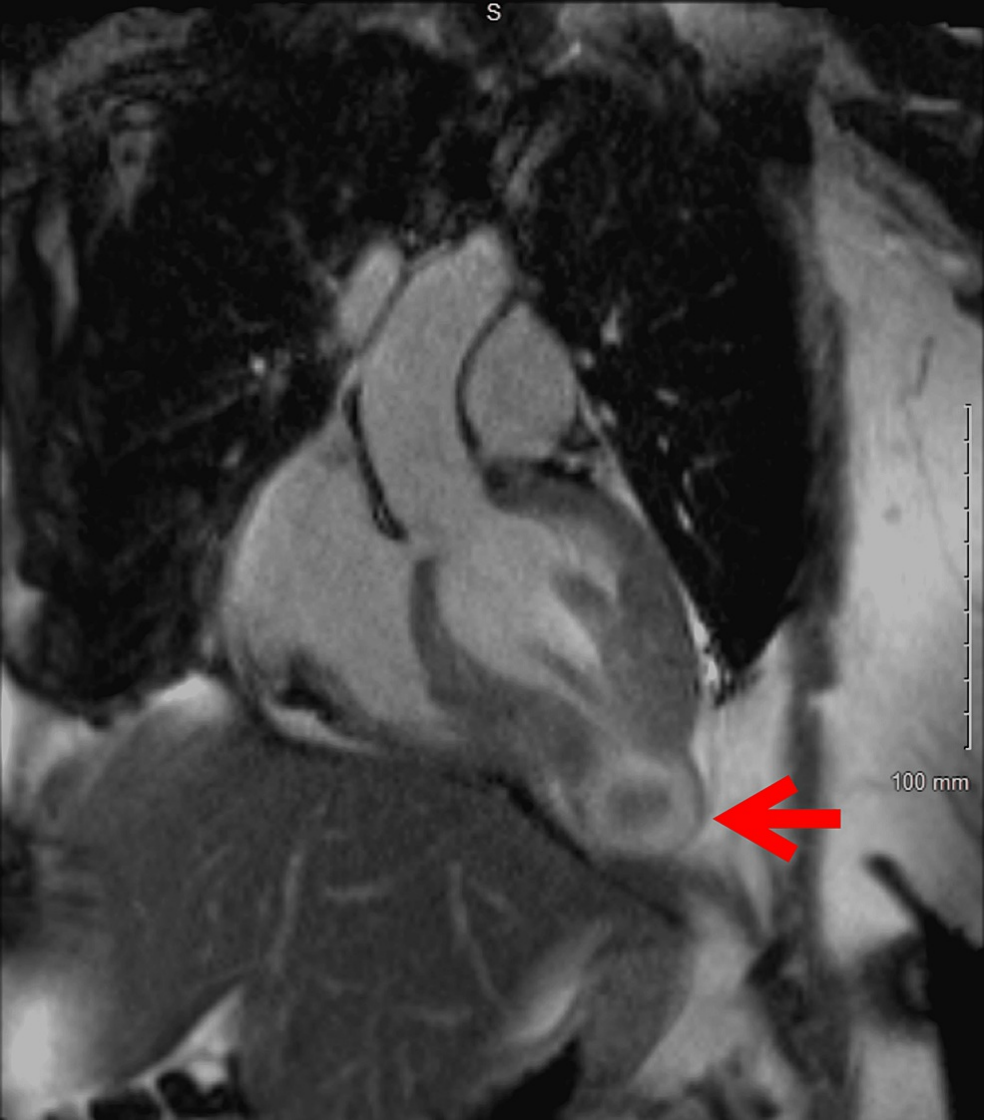
Cardiac MRI vertical long-axis view showing apical hypertrophy and an apical aneurysm A 21 x 14 mm thrombus is evident in the aneurysm, indicated with the red arrow.

**Figure 4:**
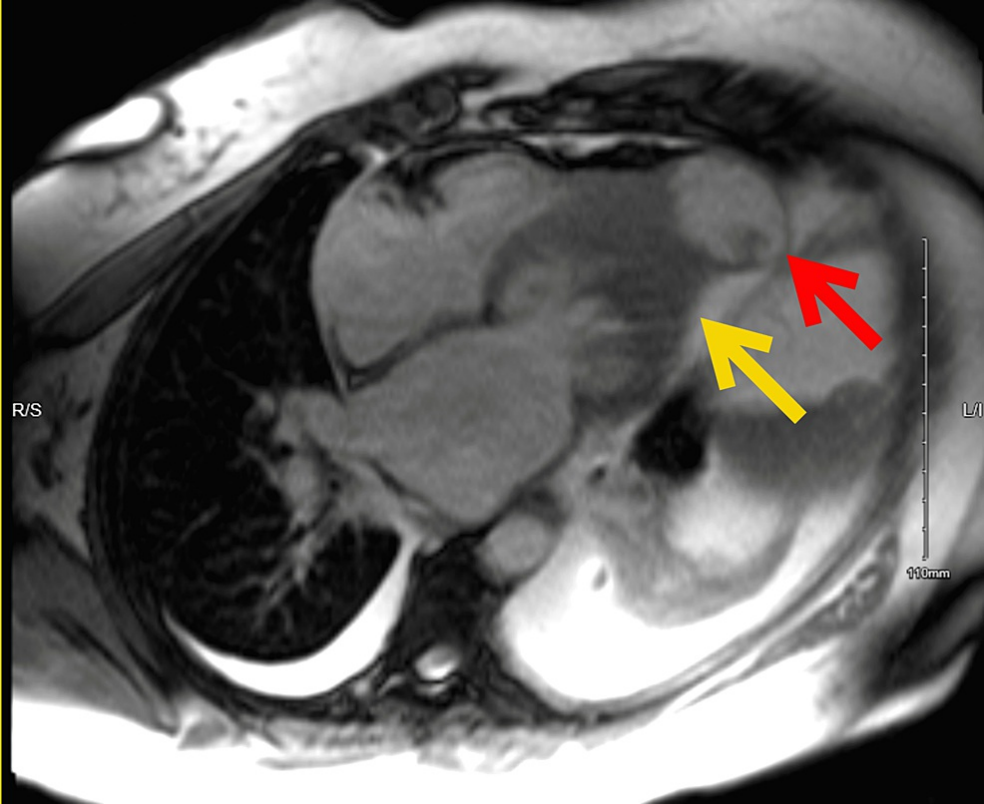
Cardiac MRI axial view showing apical hypertrophy indicated with a yellow arrow and an apical aneurysm with a 21 x 14 mm thrombus within it indicated with a red arrow

Initial EKG showed normal sinus rhythm with occasional premature ventricular complexes and a prolonged QTc interval (Figure 1). Repeat EKG while admitted showed monomorphic ventricular tachycardia (Figure 2). Chest X-ray demonstrated cardiomegaly. The initial troponin T level was 0.011 ng/mL with serial measurements up-trending to a peak value of 0.568 ng/mL. The Pro B-type natriuretic peptide (Pro-BNP) level was 1196 pg/mL. Electrolytes were within normal limits. Urine toxicology was positive for cocaine, with no quantitative levels. Left heart catheterization demonstrated normal coronaries and no vasospasm. Transthoracic echocardiogram (TTE) demonstrated apHCM, possible apical aneurysm, left atrial dilation, and a left ventricular ejection fraction (LVEF) of approximately 65%. Cardiac magnetic resonance (cMRI) imaging revealed left ventricular (LV) dyskinesia with apical aneurysm, a 21 x 14 mm thrombus, transmural late enhancement, and apical wall thickening measuring 21 mm (Figures 3-4), not showing any endomyocardial fibrosis. A detailed retrospective review of this patient’s apHCM noted that she had consistent outpatient cardiology follow-up. She was diagnosed with apHCM 12 years prior to presentation, at the age of 44. At the time, echocardiography (ECHO) displayed apical hypertrophy in a “spade” morphology, left ventricular cavity obliteration, and no evidence of an aneurysm. Periodic TTEs were performed every two years, revealing worsening apical hypertrophy but no aneurysm or thrombus formation. The most recent pre-presentation TTE was four years ago, at 52 years old. The study identified severe apical hypertrophy, a 26 mmHg gradient, and no evidence of an aneurysm. Previous Holter studies for complaints of palpitations demonstrated episodes of premature ventricular complexes (PVCs) and non-sustained ventricular tachycardia (NSVT). Prior to the coronavirus disease 2019 (COVID-19) pandemic, she was medically managed with beta-blockers and abstained from cocaine use. However, she was lost to follow-up and restarted the use of cocaine.

Treatment

The patient had successful implantation of a single-chamber defibrillator and was discharged with medical therapy for her baseline comorbidities, anticoagulation for her left ventricular thrombus, as well as amiodarone for prevention of recurrent ventricular arrhythmias. 

## Discussion

From this case report, we intend to share our experience with ApHCM, its diagnosis, sequelae, and management techniques that treated and prevented mortality secondary to this rare cardiomyopathy. ApHCM is the least common among the variants of HCM, accounting for approximately 8% of HCM [3]. Once characterized as a relatively benign structural anomaly [4], it carries risks of myocardial scarring, aneurysm, thrombosis, ventricular arrhythmias, and sudden cardiac death with an annual cardiac death rate of 0.5-4% [3]. Our case exemplifies the full spectrum of consequences secondary to apHCM their presentation and diagnosis.

This patient was diagnosed with apHCM 12 years prior to presentation, at the age of 44. At that time, ECHO displayed apical hypertrophy in a “spade” morphology, left ventricular cavity obliteration, and no evidence of an aneurysm. The American Heart Association (AHA) criteria for the diagnosis of apHCM are an apical wall thickness >15 mm and a ratio of maximal apical to posterior wall thickness greater than 1.5 on ECHO or cMRI [4]. Prior to the presentation, she had consistent outpatient cardiology follow-ups. Periodic TTEs were performed every two years, revealing worsening apical hypertrophy but no aneurysm or thrombus formation. The most recent pre-presentation TTE was four years ago, at 52 years old, which identified severe apical hypertrophy, a 26 mmHg gradient, and no evidence of aneurysm. The only other chronological documentation of apical aneurysm formation in a patient with apHCM is by Kawai et al. [5], suggesting that high apical systolic flow velocities of >1.5 m/s in apHCM may anticipate aneurysmal formation. Chronic apical stress predisposes patients to aneurysm formation, affecting 13-15% of apHCM cases [3] and is associated with significant morbidity and mortality. Since the last outpatient echocardiogram four years ago, untreated apical stress likely prompted the development of a left ventricular (LV) aneurysm in our patient. Novel techniques like guidewire measurement of high intracavitary pressure gradients have been proposed as a possible diagnostic tool for myocardial degeneration, aneurysm formation, and arrhythmogenic changes in apHCM [6]. Medical management for apHCM and aneurysm prevention are uniform, constituting beta-blockers as a first-line option and non-dihydropyridine calcium channel blockers as the second-line option [3]. Heart transplant is the gold standard treatment for the failure of medical management in HCM. Recently, transapical myomectomy, a unique procedure, has shown to provide significant clinical improvement [7-8], with long-term survival better than candidates awaiting heart transplants. The 10-year survival was 80% in the myomectomy group versus 63% in patients awaiting a transplant on medical therapy [9]. Comparisons to heart transplants have yet to be established. Minimally invasive options of alcohol septal ablation did not significantly improve stroke volume.

During this patient’s hospitalization, she developed a VT storm, precipitated by a reentry circuit around the aneurysm. She initially failed chemical cardioversion, became hemodynamically unstable, and attempted electrical cardioversion was unsuccessful. She was intubated, mechanically ventilated, sedated on high-dose propofol, and started on a beta-blocker. The above management has been proven efficacious in a VT storm in the setting of acute myocardial infarction by decreasing the sympathetic tone [10], however, its effectiveness is unknown in a VT storm precipitated by aneurysmal reentrant circuits in the setting of non-ischemic cardiomyopathies. Despite all efforts to resolve the challenging arrhythmia, the patient suffered a cardiac arrest and required cardiopulmonary resuscitation.

Regarding the differential diagnosis of apHCM, our patient’s cMRI showed the pathognomonic “spade” shape, a new apical aneurysm, and a 21 x 14mm thrombus with late enhancement in the apical cavity and apex shown in Figures 3-4, epitomizing all the rare and severe consequences of apHCM. Differentials such as hypertrabeculation, non-compaction, and endomyocardial fibrosis (EMF) were excluded by cMRI using LGE. At the cellular level, cardiac myocyte hypertrophy and its contraction results in microvascular obstruction and therefore perfusion defects, commonly presenting as chest pain, dyspnea, and decreased exercise tolerance [11]. Our patient was initially evaluated for the acute coronary syndrome (ACS) with typical chest pain in the setting of multiple known risk factors. Ischemia workup was negative, however, cMRI appreciated transmural late enhancement and increased edema compatible with distal left anterior descending (LAD) infarction. This is due to the aforementioned mechanism of increased contractility of hypertrophied myocytes in the apex, compressing the coronary vascularate, correlating with the ischemia and inflammatory changes in the territory of distal LAD with clear coronaries.

Left ventricular thrombi (LVT) are more commonly reported with ischemic cardiomyopathies, however, non-ischemic cardiomyopathies (NICM) should also be considered. The prognosis of patients is poor, with a high risk of mural thrombus embolism OR 5.45 (95% CI 3.02-9.83) [12]. Anticoagulation with vitamin K antagonists is the standard of care, however, similar rates of LVT resolution and major adverse cardiac events (MACE) have been observed with the off-label use of direct oral anticoagulants (DOACs). Current guidelines for LVT treatment and duration were established on ST-elevation myocardial infarction (STEMI) as the etiology of LVT. The American College of Cardiology (ACC) Foundation (ACCF) gives a Class IIa recommendation of VKA treatment for three months. There are no specific guidelines for the treatment of nonischemic causes of LVT. Predictors of LVT in cases of NICM have been identified as LVEF, LGE a marker of myocardial injury, and LGE extent on cMRI [13]. LGE was evident in our patient's cMRI albeit during LVT visualization.

Hypertrophic cardiomyopathy, being the most prevalent genetic cardiac disease, with an elevated risk of sudden cardiac death, warrants the consideration of screening echocardiography and genetic testing. Hundreds of gene mutations encoding sarcomeres have been established as a cause of HCM. Echocardiography-guided genetic testing yielded a 30% myofilament gene positivity [13]. To date, the recommendation is to genetically test the initial case if a mutation is confirmed, annual echocardiography, and possibly genetic testing in first-degree relatives [14-15]. In our patient, who did not have genetic testing, interval echocardiography of the initial case and screening of first-degree relatives with echocardiogram and electrocardiogram (EKG) are advocated.

## Conclusions

Hypertrophic cardiomyopathy (HCM) is the most prevalent genetic cardiac disease. Recognition of apical hypertrophic cardiomyopathy (apHCM) is critical in preventing progressive remodeling, predisposing to aneurysms, reentrant circuits, mural thrombus formation, and cardiac arrest. We hope that upon diagnosis, medical and/or surgical management and close follow-up can be standardized to prevent the aforementioned consequences.
